# Estimating Costs of Implementing Stroke Systems of Care and Data-Driven Improvements in the Paul Coverdell National Acute Stroke Program

**DOI:** 10.5888/pcd16.190061

**Published:** 2019-10-03

**Authors:** Benjamin Yarnoff, Olga Khavjou, Joanna Elmi, Kincaid Lowe-Beasley, Christina Bradley, Jacqueline Amoozegar, Devon Wachtmeister, Janice Tzeng, John McCoy Chapel, Stephanie Teixeira-Poit

**Affiliations:** 1 RTI International, Public Health Economics Program, Research Triangle Park, North Carolina; 2 Centers for Disease Control and Prevention, Division of Heart Disease and Stroke Prevention, Atlanta, Georgia; 3 North Carolina Agricultural and Technical State University, College of Health and Human Sciences, Greensboro, North Carolina

## Abstract

**Purpose and Objectives:**

We evaluated the costs of implementing coordinated systems of stroke care by state health departments from 2012 through 2015 to help policy makers and planners gain a sense of the potential return on investments in establishing a stroke care quality improvement (QI) program.

**Intervention Approach:**

State health departments funded by the Paul Coverdell National Acute Stroke Program (PCNASP) implemented activities to support the start and proficient use of hospital stroke registries statewide and coordinate data-driven QI efforts. These efforts were aimed at improving the treatment and transition of stroke patients from prehospital emergency medical services (EMS) to in-hospital care and postacute care facilities. Health departments provided technical assistance and data to support hospitals, EMS agencies, and posthospital care agencies to carry out small, rapid, incremental QI efforts to produce more effective and efficient stroke care practices.

**Evaluation Methods:**

Six of the 11 PCNASP-funded state health departments in the United States volunteered to collect and report programmatic costs associated with implementing the components of stroke systems of care. Six health departments reported costs paid directly by Centers for Disease Control and Prevention–provided funds, 5 also reported their own in-kind contributions, and 4 compiled data from a sample of their partners’ estimated costs of resources, such as staff time, involved in program implementation. Costs were analyzed separately for PCNASP-funded expenditures and in-kind contributions by the health department by resource category and program activity. In-kind contributions by partners were also analyzed separately.

**Results:**

PCNASP-funded expenditures ranged from $790,123 to $1,298,160 across the 6 health departments for the 3-year funding period. In-kind contributions ranged from $5,805 to $1,394,097. Partner contributions (n = 22) ranged from $3,912 to $362,868.

**Implications for Public Health:**

Our evaluation reports costs for multiple state health departments and their partners for implementing components of stroke systems of care in the United States. Although there are limitations, our findings represent key estimates that can guide future program planning and efforts to achieve sustainability.

**Summary**

**What is already known on this topic?**

Previous studies examined the costs and cost-effectiveness of stroke care quality improvement (QI) interventions at the clinical level, but nothing is known about QI initiatives that span the stroke system of care.

**What is added by this report?**

The Paul Coverdell National Acute Stroke Program (PCNASP) is a stroke QI initiative that spans the full stroke system of care. This study examined the costs of implementing PCNASP QI initiatives.

**What are the implications for public health practice?**

Findings can guide future stroke program planning, QI, and efforts to achieve sustainability.

## Introduction

Each year, approximately 800,000 people in the United States have a stroke, and 1 American will die from a stroke every 4 minutes (1). Stroke is also a leading cause of serious long-term disability (1). Previous research and stroke guidelines show that the use of stroke systems of care and stroke care quality improvement (QI) programs can improve health outcomes (2–15). A systems-based approach to stroke care promotes the coordination of services along the continuum of prevention and care, that is, primary prevention, community education, activation of emergency medical services (EMS), acute stroke treatments, secondary prevention, rehabilitation, reintegration with community, and continuous QI activities at each stage of care (5). Once effectiveness is established, understanding the resources needed to complete activities is essential for program planning and implementation, the sustainability of future QI initiatives, and potential program replication.

The Centers for Disease Control and Prevention (CDC) contracted with RTI International, a nonprofit research institute, to conduct an independent evaluation of state health departments funded by the Paul Coverdell National Acute Stroke Program (PCNASP), during 2012 through 2015. The evaluation included an exploration of costs incurred to implement program activities. Previous studies documented the impact of the PCNASP on stroke outcomes and process measures (16,17); however, to provide information on stroke program implementation for public health departments, this study aimed to estimate direct activity-based costs and in-kind contributions of executing a stroke systems of care program that includes an estimate of partner contributions toward achieving shared objectives of the PCNASP.

## Purpose and Objectives

At the direction of Congress, CDC began the PCNASP in 2001 to develop prototype stroke registries and to measure and improve the quality of stroke care (18). The 2012–2015 PCNASP cooperative agreement provided “funds to support and strengthen the capacity and leadership of state health department’s heart disease and stroke prevention program . . . [to improve] acute stroke treatment and outcomes through the implementation of Paul Coverdell Acute Stroke registries” (18). PCNASP is unique because it funds state health departments to convene strategic partnerships and guide implementation of program strategies that use elements of a patient-level disease registry in driving stroke care QI across stroke treatment settings. The focus on expanding QI interventions to improve delivery of evidence-based clinical interventions reflects a multilevel strategy aimed at improving patient care and advancing population health. In 2012, CDC funded 11 state health departments through 3-year cooperative agreements to improve the quality of stroke care in various settings across the continuum of care: 1) in-hospital care only, 2) in prehospital care and in-hospital care, 3) in posthospital care, or 4) in all 3 settings.

Many studies have examined the costs and cost-effectiveness of stroke care QI interventions at the clinical level (19–31). The unique goal of PCNASP is to use the resources provided by CDC to state health departments to catalyze QI in stroke systems of care. Only a few examples of small programs exist where costs were analyzed and where a central entity, such as a health department, catalyzed hospital-level QI. For example, a stroke telemedicine initiative in New York was estimated to cost $20,000 to $26,134 per year for each participating hospital (32), and a study in England implementing stroke prevention guidelines in primary care practices found a cost of ₤1,500 ($2,959 USD) per practice (33). However, both of these estimates were for selected aspects of stroke care only and did not include a comprehensive view of the costs of program implementation; they also did not capture data on the costs needed from a state public health department perspective. A study of the costs incurred by state health departments for implementing a registry for cancer patients found it cost $93.11 per case for state health departments with a low volume of cases (<10,000) and $27.70 per case for state health departments with a high volume (>50,000) (34). However, the purpose of cancer registries is to serve as a census of all cancer cases in the state, but the purpose of PCNASP is to use a registry-based approach to catalyze data-driven QI for stroke care. Although studies have documented costs of QI initiatives or disease registry programs, information is limited on programs analogous to PCNASP and focused on developing stroke systems of care from a state health department perspective. Studies have documented the progress of PCNASP at the federal program level and within a state-funded program. During 2008 through 2013, PCNASP demonstrated improvements in key quality measures of stroke care such as a 40 percentage-point increase in treatment with intravenous tissue-type plasminogen activator within 45 minutes (ie, door-to-needle-time) (16). One state-led program demonstrated an association between quality of care measures and stroke mortality among their state’s participating hospitals. For example, mortality at hospitals with low-quality care was 294% higher than at hospitals with high-quality care, and mortality at hospitals with intermediate-quality care was 38% higher (17).

In addition to documenting costs to state health departments, capturing costs of resources from partners is key to understanding costs of program implementation. Recent studies examining the costs of large public health grants reported that in-kind contributions of partner organizations might constitute a large portion of total program costs (35). Previously, an activity-based costing approach was used to examine state cancer registries that operate similarly to state stroke care registries (34,36,37) and other public health programs (35) and is recommended for use in strategic planning (38).

Understanding the costs of implementing the components of stroke systems of care and conducting data-driven QI is essential for future program planning, because this knowledge will help decision makers to ensure that stroke care QI programs are funded appropriately and resources are allocated wisely. Quantifying in-kind partner organization use of resources is vital to program planning and understanding the true value of partner contributions.

## Intervention Approach

**Figure 1:**
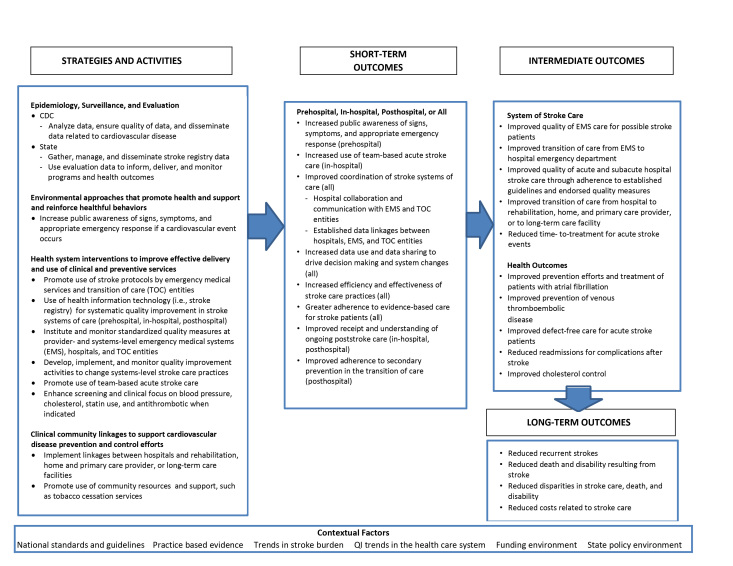
Logic model for Paul Coverdell National Acute Stroke Program.

State health departments funded by PCNASP received CDC funding to implement activities to support the start and proficient use of hospital stroke registries statewide and to coordinate data-driven QI efforts aimed at improving the treatment and transition of stroke patients across the continuum of care settings, from prehospital EMS to in-hospital care to postacute care facilities. Health departments provided technical assistance and data to support hospitals, EMS agencies, and posthospital care agencies to undertake small, rapid, incremental QI efforts to produce more effective and efficient stroke care practices. A program logic model, describing strategies and activities, contextual factors, and anticipated short-term, intermediate, and long-term outcomes was developed (Figure 1).

CDC funded state health departments to “work collaboratively with public and private partners to implement components of an integrated stroke system of care with a strong focus on QI and effective and efficient transitions of care for stroke patients” (18). To achieve this goal, health departments partnered with EMS agencies, hospitals, postacute care facilities, and medical and public health associations, such as the American Heart Association/American Stroke Association, among others. PCNASP-funded state health departments were required to form an advisory group or steering committee to guide their efforts, with representation from state stakeholders, including stroke care professionals, EMS personnel, coordinators of stroke care, and department of health staff members. Cost sharing and matching funds from state health departments were not required.

From 2012 through 2015, PCNASP-funded state health departments implemented a wide variety of stroke care QI activities across the continuum of care. Activities included webinars, learning collaboratives, regional workshops, communication tools development to promote feedback, and best practice sharing across stroke care settings. Additionally, they monitored and regularly provided technical assistance to hospitals collecting a set of standardized performance measures. These stroke care and outcome performance measures determined the content and focus areas of the hospital-specific QI activities, as required by the CDC Funding Opportunity Announcement DP12-1203 (18). Table 1 provides a brief summary of key program characteristics and QI activities of the 6 PCNASP-funded state health departments that participated in the cost study. The health departments supported improvements in stroke care at an average of 50 partner hospitals per state, which in turn, provided care for an average of 79.4% of annual statewide stroke admissions. QI activities ranged across health departments and show the diversity of the approaches taken by the health departments.

**Table 1:** Key Characteristics and Quality Improvement Activities of the 6 Paul Coverdell National Acute Stroke Program (PCNASP) State Health Departments, 2012–2015

State	Continuum of Care Settings (Maximum = 3)	No. of PCNASP- Funded FTEs^a^	No. of PCNASP Partner Hospitals^a^	Annual % Statewide Stroke Admissions for PCNASP Partner Hospitals^b^	Quality Improvement Activities Facilitated by Health Department
**A**	2	4.20	42	43.4	• Provided training and technical assistance for hospital staff and EMS responders • Established pilot program to improve EMS-to-hospital transitions of care • Conducted program reviews of performance measures with hospital staff • Provided awards for hospitals that achieved excellence
**B**	2	3.15	67	86.3	• Supported QI through EMS feedback • Provided training on ASLS and data abstraction • Promoted communication with hospitals through collaborative planning activities • Presented bimonthly QI webinars • Supported QI through performance improvement reports for hospitals
**C**	3	2.30	51	83.5	• Held EMS Stroke QI Collaborative regional meetings, hospital regional meetings, and Postacute Stroke Care Collaborative regional meetings • Provided QI technical assistance calls and QI coaching at site visits • Provided data trainings • Developed hospital communication form to provide feedback to EMS • Provided individual and aggregate cross-hospital performance reports • Provided postacute care education on the signs and symptoms of stroke and need to call EMS for suspected strokes
**D**	2	2.75	61	90.2	• Conducted regional education workshops, quarterly conference calls, and performance improvement collaborative meetings • Implemented performance improvement projects • Provided technical assistance and conducted site visits • Conducted research on use of community paramedics in transition from hospital to home care
**E**	2	1.0	51	89.5	• Hosted bimonthly learning webinars and regional education workshops • Implemented QI grants • Supported networking through listservs and stroke coordinators mentor list
**F**	2	1.30	29	83.3	• Developed EMS-to-hospital transfer protocol for patients who receive alteplase to ensure routing to designated hospital • Developed hospital communication form to provide feedback to EMS • Developed 2 QI toolkits on how to build a stroke program and EMS resources • Conducted site visits to deliver technical assistance on data re-abstraction and monitoring of performance measures • Provided hospitals with quarterly QI reports • Hosted educational outreach events on best practices
**All states, mean**	2.2	2.4	50	79.4	—

Abbreviations: ASLS, advanced stroke life support; CDC, Centers for Disease Control and Prevention; EMS, emergency medical services; FTE, full-time employee; QI, quality improvement.

a As reported in 2015 PCNASP final reports.

b The number of statewide stroke admissions was based on 2014–2015 data in 2015 PCNASP final reports. Methods of calculation and reporting varied across states.

## Evaluation Methods

In June 2015, of the 11 PCNASP-funded health departments, 6 volunteered to collect and report data on programmatic costs associated with implementing the components of stroke systems of care using a data collection instrument based on Excel (Microsoft Corporation) and designed for required activities of the PCNASP. Nine health departments were eligible to participate in the study because they met the following 2 criteria: 1) they were working in at least 2 stroke care settings; and 2) their programs were fully implemented. Of the 6 participating health departments, 5 were funded to focus exclusively on prehospital and in-hospital stroke care, and one was funded to focus on prehospital and in-hospital stroke care and transition to posthospital settings. Four of the 6 health departments had participated in previous rounds of PCNASP. Newly funded health departments did not differ in any observable way from the health departments that had been funded previously. These 6 state health departments partnered with a total of 467 hospital, EMS, and nonclinical organizations in their states, ranging from 37 to 125 partners per state.

The data collection tool was designed to capture data on implementation costs of the state health departments and their partners from a public health system perspective. The tool used an activity-based approach to allocate costs across 6 primary program activities: data collection, data linkage across settings, and data management; clinical guidance and expertise; QI; building and maintaining partnerships; program evaluation; and administration. Participating health departments reported costs paid directly by CDC-provided funds, and 5 also reported their own in-kind contributions. In-kind contributions were defined as costs of resources used by the health department as part of its work on the PCNASP but not paid with PCNASP funds, such as staff members funded by internal state funds or other grants and volunteers. Four of the 6 state health departments voluntarily collected and compiled data from a sample of their partners on the estimated costs of resources, such as staff time involved in program implementation as part of their participation in PCNASP. These costs were not paid through the cooperative agreement. Rather, they represent the value of the partners’ resources used to implement components of stroke systems of care as part of their engagement with PCNASP.

PCNASP program administrators in each participating state health department completed the data collection instrument, and an economist from the study team was available throughout data collection to provide technical assistance. Program administrators reported the actual expenditures on resources used for program implementation during the 3-year cooperative agreement. The data collection instrument was used to collect information on the programmatic costs for 4 resource categories: 1) labor; 2) contracts; 3) materials, travel, equipment, and services; and 4) indirect costs. The labor category included a list of staff member roles working on the PCNASP at any time during the funding period, their wages plus fringe benefits, the number of months they worked on the PCNASP, and the average fraction of time they spent working on the PCNASP. This was used to generate the total cost of each staff member to the PCNASP during the 3 years. The contracts category included a list of all contracts funded by the program, such as consultants providing support for QI and the total amount of the contracts. Respondents reported data on materials, travel, equipment, and services, which included expenditures such as funds for software to support data collection, analysis, and travel for training. Finally, the data collection tool requested the total amount of indirect costs (ie, overhead costs that are not directly attributable to 1 project, such as facility expenditures) charged to the PCNASP during the 3 years.

The data collection instrument asked respondents to provide estimates of the percentage of each resource, such as a staff member, equipment, and a consultant, devoted to each of the 6 primary program activities. Data were collected in June 2015, the last month of the cooperative agreement, and respondents were asked to report allocations for each activity that was representative of the entire 3-year funding period.

The data collection instrument contained a component for the state health departments to provide information on contributions of partners. Health departments were to include only the contributions of partners associated with stroke QI and only from a convenience sample of 10% of their partners to minimize data collection burden. Partners were asked to exclude costs related to any stroke QI initiatives other than PCNASP. Program administrators in 4 states entered data from a convenience sample subset of their partners (n = 22, 5% of all partners in the 6 states). These 22 partners were not representative of all PCNASP partners. Participating partners provided data for labor and nonlabor resources and the allocation of each resource across key program activities, similar to the data provided by the state health departments on their expenditures. The convenience sample included 9 large hospitals, 8 small hospitals, and 5 nonhospital organizations across all reporting states. We defined large hospitals as those with an annual caseload (number of patients seen in a year) of more than 20,000, and small hospitals were defined as those with an annual caseload of 20,000 or fewer. The average annual number of stroke patients seen in the sample of partner hospitals was 523 for large hospitals and 337 for small hospitals. Nonhospital organizations included medical and public health associations. We examined costs separately for large hospitals, small hospitals, and nonhospital organizations.

Costs were analyzed separately for PCNASP-funded expenditures, in-kind contributions by the health department, and in-kind contributions by partners. Within PCNASP-funded expenditures and in-kind contributions by the health department, we examined costs by resource category (labor, contracts, materials, travel, equipment, services, and indirect costs for PCNASP-funded expenditures, and labor and nonlabor for in-kind contributions) and by programmatic activity. Costs for each resource category were constructed as the cost of each expenditure multiplied by the estimated fraction of that expenditure used for the PCNASP. To construct labor costs, each staff member’s total salary was multiplied by the percentage of their salaried time spent working on the PCNASP. The study team constructed costs for each activity by multiplying expenditures by their estimated percentage allocated to that activity. Data were collected retrospectively at 1 time point representing the 2012–2015 program period; therefore, it was necessary to make 2 assumptions: 1) the reported percentage allocated to PCNASP activities was representative of the entire funding period, and 2) staff salaries were constant during the funding period.

## Results

PCNASP-funded expenditures during the funding period ranged from $790,123 to $1,298,160; much of these costs were for labor, but some health departments had substantial expenditures on contracts (Table 2). Labor generally included staff members to manage program activities, such as project coordinators and program directors. Some grantees included QI and information technology (IT) specialists. Grantees that did not directly employ QI or IT specialists typically contracted for those services. More than one-half of PCNASP funds were dedicated to supporting an average of 2.45 full-time employees at a state health department. Overall, expenditures for labor were lower in health departments in which contract expenditures were high. For 4 of the 6 health departments, QI was the activity that incurred the greatest expense. Slightly more than one-third of total expenditures were devoted to QI. The remaining activities did not have a consistent pattern across the health departments.

**Table 2:** Implementation Costs for 6 State Health Departments in the Paul Coverdell National Acute Stroke Program (PCNASP), 2012–2015^a^

Cost Metric	State A	State B	State C	State D	State E	State F	Median
**3-year PCNASP-funded expenditures, $**	944,910	1,030,347	1,298,160	930,964	790,123	954,791	949,850
**PCNASP-funded expenditures, by resource category, $**							
Labor	746,952 (79%)	489,908 (48%)	516,380 (40%)	669,266 (72%)	200,195 (25%)	139,394 (15%)	503,144
Contracts	57,484 (6%)	223,532 (22%)	655,341 (50%)	42,226 (5%)	472,411 (60%)	728,503 (76%)	347,972
Materials, travel, services, and equipment	4,600 (0.5%)	254,238 (25%)	80,986 (6%)	44,672 (5%)	51,064 (6%)	79,342 (8%)	65,203
Indirect	135,874 (14%)	62,670 (6%)	45,453 (4%)	174,800 (19%)	66,453 (8%)	7,552 (1%)	64,561
**PCNASP-funded expenditures, by activity, $**							
Data collection, linkage, and management	155,404 (16%)	209,446 (20%)	19,500 (2%)	178,615 (19%)	95,191 (12%)	68,150 (7%)	125,298
Clinical guidance and expertise	134,365 (14%)	196,812 (19%)	63,725 (5%)	68,192 (7%)	35,622 (5%)	102,409 (11%)	85,300
Quality improvement	303,763 (32%)	368,024 (36%)	615,982 (47%)	159,327 (17%)	519,814 (66%)	211,120 (22%)	335,894
Building and maintaining partnerships	123,316 (13%)	20,967 (2%)	123,305 (9%)	96,165 (10%)	25,546 (3%)	221,869 (23%)	109,735
Evaluation	73,626 (8%)	76,556 (7%)	193,874 (15%)	65,361 (7%)	23,621 (3%)	140,430 (15%)	75,091
Administration	154,436 (16%)	158,542 (15%)	281,774 (22%)	363,304 (39%)	90,330 (11%)	210,812 (22%)	184,677
**Health department in-kind contributions, $**	5,805	846,737	6,833	0	1,394,097	5,825	6,329
By resource category, $							
Labor	1,763 (30%)	201,043 (24%)	6,833 (100%)	0	159,592 (11%)	5,825 (100%)	6,329
Nonlabor	4,042 (70%)	645,694 (76%)	0	0	1,234,505 (89%)	0	2,021
By activity, $							
Data collection, linkage, and management	3,018 (52%)	402,161 (47%)	0	0	234,000 (17%)	756 (13%)	1,887
Clinical guidance and expertise	818 (14%)	107,981 (13%)	0	0	58,500 (4%)	756 (13%)	787
Quality improvement	875 (15%)	90,941 (11%)	0	0	786,940 (56%)	0	438
Building and maintaining partnerships	875 (15%)	18,617 (2%)	1,708 (25%)	0	160,315 (11%)	461 (8%)	1,292
Evaluation	57 (1%)	7,080 (1%)	1,708 (25%)	0	84,698 (6%)	791 (14%)	1,250
Administration	162 (3%)	219,957 (26%)	3,416 (50%)	0	69,644 (5%)	3,060 (53%)	3,238

a Percentages sum to 100% along columns for each category.

Distribution of in-kind contributions from state health departments was bimodal. Four health departments used less than $7,000 in in-kind contributions during the funding period, with 1 health department reporting no in-kind contributions. Two health departments reported substantial in-kind contributions ($394,097 and $846,737). In those 2 health departments, most costs were for nonlabor resources. Both of these health departments contributed a large amount of in-kind funds on data collection, linkage, and management, although only one of them focused most of its in-kind contributions on QI.

**Figure 2:**
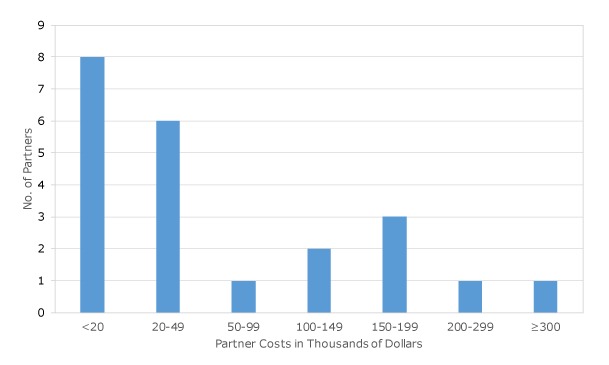
Spending among partners in the Paul Coverdell National Acute Stroke Program.

Four of the 6 PCNASP-funded state health departments reported data from a convenience sample subset of 22 partners, representing the estimated resources to implement components of stroke systems of care as part of their engagement with PCNASP (Table 3). The average value of partner resources was $75,295 per partner (large hospital, $133,399; small hospital, $22,161; nonhospital, $55,722), but as with most cost data, the distribution was somewhat skewed, and the median amount was $29,049 (large hospital, $118,757; small hospital, $7,071; nonhospital, $17,533). Partner resources ranged from $3,912 for 1 small hospital to $362,868 for 1 large hospital. Differences in the amount of partner resources aligns with differences in partner organization type (hospital and nonhospital) and size (large hospitals and small hospitals). Large hospital partners had a median resource value of $118,757, and small hospital partners had a median resource value of $7,071. Nonhospital organizations had a median resource value of $17,533. Most partners spent less than $50,000 in resources during the study period, but some spent substantially more (Figure 2). The sample of 22 partners, however, represents only a small portion of the 467 partners (4.7%) with whom PCNASP-funded state health departments worked.

**Table 3:** Summary of Costs for Selected Partners in the Paul Coverdell National Acute Stroke Program (PCNASP) in 4 Participating States, 2012–2015

Type of Partner	No. of Partners	Average Cost, $	Median Cost, $	Minimum Cost, $	Maximum Cost, $
Large hospital	9	133,399	118,757	25,039	362,868
Small hospital	8	22,161	7,071	3,912	96,727
Nonhospital organization^a^	5	55,722	17,533	7,349	213,289
Total	22	75,295	29,049	3,912	362,868

a Nonhospital organizations include medical and public health organizations.

**Table:** 

Partner Costs, $	No. of Partners
<20,000	8
20,000-49,000	6
50,000-99,000	1
100,000-149,000	2
150,000-199,000	3
200,000-299,000	1
≥300,000	1

## Implications for Public Health

This study quantified preliminary estimates of 3 areas of costs for implementing 2 or more components of a stroke system of care and related QI activities from a state public health perspective. Cost areas included activity-based PCNASP-funded expenditures of the state health department, in-kind contributions by health departments, and resources used by partners. Previous studies demonstrated the costs and effectiveness of certain clinical interventions for stroke care, but little was known about the costs incurred to implement a systems-level program such as PCNASP, which supported hospitals in delivering these evidence-based clinical interventions to patients with stroke. Documenting programmatic costs is an important step toward building the evidence base for public health approaches to health care delivery for acute stroke care.

Findings related to PCNASP-funded expenditures indicated that most PCNASP funds were dedicated to supporting an average of 2.45 full-time employees working at the state health department. When all programmatic activities were considered, this study indicated that approximately one-third of PCNASP expenditures were used to coordinate and support QI efforts. With relatively limited resources, the 6 PCNASP-funded state health departments supported improvements in stroke care at an average of 50 partner hospitals per state, which in turn, provided care for an average of 79.37% of annual statewide stroke admissions. Results showed differences across levels of PCNASP-funded expenditures ($790,123 to $1,298,160 during the 3-year period), a mix of resources (some programs relied heavily on state health department labor and others relied heavily on consultants), and level of expenditures on program activities. Four of the 6 state health departments devoted more PCNASP funding to QI than to any other activity. Indirect costs were relatively low across grantees, which might not be the case in other types of organizations, such as nongovernmental organizations or hospitals, and speaks to the value of working through state health departments to bridge public health and clinical systems to improve stroke care. This wide range of expenditures for program implementation is consistent with the diversity of approaches that PCNASP-funded state health departments took from 2012 through 2015 to advance stroke systems of care across care settings. For example, State E had the highest cost and primarily implemented actual grants for QI and facilitated learning platforms (Table 1). A large percentage of State E costs were in-kind, indicating that it was able to leverage other funding streams for implementation. In contrast, State D had the lowest cost and took an approach of providing technical assistance for QI. State D also did not use any in-kind contributions for program implementation (Table 2). These extremes highlight alternative forms of implementation and their cost implications. Showing a range of implementation possibilities can help encourage and support future program decisions. Additionally, 4 of the 6 grantees that participated in the cost study received PCNASP funds before the 2012–2015 program, and programmatic costs might vary by stage of program implementation. The composition of grant-funded expenditures should be documented to gain a better understanding of how implementation of the PCNASP supports health system-level QI to improve patient outcomes. State health departments are positioned to serve as a keystone for supporting statewide stroke systems of care, and it is not surprising that findings from this study show that most program costs were incurred to support staff and QI.

In-kind contributions are key to define because program planners might underestimate the importance of these costs (35). Our study found that in-kind contributions from health departments varied from negligible to substantial. Four of the 6 state health departments participating in this study reported in-kind contributions of less than $7,000. This lack of funding from in-kind contributions underscores the importance of providing PCNASP funding to health departments for establishing stroke systems of care and implementing QI; without PCNASP funding, these 4 health departments would not have been able to leverage the internal resources needed to implement components of stroke systems of care. In contrast, 2 health departments contributed $1,394,097 and $846,737 in-kind. Most of these in-kind contributions were for nonlabor costs, which indicates that these health departments used funds for large purchases of materials, equipment, or contracted services that were above and beyond CDC PCNASP funding. The 2 grantees that had large in-kind contributions received previous PCNASP funds before the 2012–2015 program. However, the other 2 previously funded grantees used similar amounts of in-kind contributions to those of grantees that had not participated before the 2012–2015 program. As programs become more established, it is possible that they are better able to leverage CDC PCNASP funding for additional support in their stroke registry and QI efforts. Overall, in-kind contributions are an essential point for planners to consider when designing program implementation. That some state health departments made large in-kind contributions to meet their goals demonstrates that the diversity of programs across states might require health departments to leverage PCNASP funds to obtain additional resources to achieve their goals.

Comparing cost estimates to the published estimates of the impact of PCNASP can help policy makers and planners estimate the potential return on investments in this type of stroke QI program. A study assessing the progress of PCNASP on selected stroke quality of care measures between 2008 and 2013 demonstrated an increase in the percentage of stroke patients receiving IV alteplase by 9 percentage points; the percentage of stroke patients with a door-to-needle time less than 60 minutes by 40 percentage points; and the percentage of stroke patients with a door-to-needle time less than 45 minutes by 30 percentage points (16). Quality of stroke care delivered is associated with long-term health outcomes, as demonstrated by a state-led program in Georgia that found hospitals delivering lower quality of care had a 1-year mortality rate 294% higher than those with high-quality care, and those with intermediate-quality care had a 1-year mortality rate 38% higher than those with high-quality care (17). Across a broader body of stroke literature, IV alteplase is reported to be more effective in treating stroke patients when it is administered as close as possible to the time of onset (39). Furthermore, the timely use of IV alteplase among acute stroke patients is considered a cost-effective treatment for stroke (40) and is associated with improved long-term stroke outcomes (41). Although the literature demonstrates improvements in quality of stroke care among the PCNASP, it is vital to understand the value of the full program investment by assessing programmatic costs intended to catalyze improvements in stroke quality of care. Future studies should investigate the association between improved health care processes and outcome measures relative to the value of resources invested by the PCNASP through state health department–funded programs.

The estimated value of partner resources, such as staff time, to implement stroke systems of care was sizeable; median levels were $118,757 for large hospital partners, $7,071 for small hospital partners, and $17,533 for nonhospital partners. Although these resources were devoted to implementing stroke systems of care as part of the partnership with PCNASP, organizations might have allocated these resources to improving stroke systems of care independent of PCNASP. These estimates are likely not representative of the full sample of partners. However, if they are overestimates, summing the costs of resources used across all 467 PCNASP partners would be large, relative to total PCNASP funding. A study of CDC’s Communities Putting Prevention to Work program reported that partner costs made up a large portion of total program costs (35). In the PCNASP, hospital partners implement the QI initiatives and systems-level or practice changes onsite, such as instituting EMS and hospital protocols or policies. Therefore, it is reasonable to think that the cost of resources involved in implementation would represent a large portion of the total program costs.

PCNASP funding aims to provide “support for the development of strategic partnerships for improving stroke care at the state level and thus encourages implementation of QI activities with EMS, hospitals, stroke specialists, and rehabilitation facilities” (18). PCNASP-funded state health departments and partner organizations form a collective and leverage each other’s strengths to achieve progress and improve outcomes. Accordingly, it is crucial that partners and state health departments are aware of and have appropriate expectations for the estimated value of resources, time, and costs of program implementation to assess their ability to participate in the program and to confirm that their participation is worth the investment. In addition, program planners at the state health department might be able to craft recruitment materials and partnership agreements that communicate an estimated range of in-kind contributions that partners are likely to bring to the collaborative effort.

This study has 3 main limitations. First, data were collected retrospectively for the entire funding period, which might have contributed to recall errors and prohibited the ability to accurately determine how costs were distributed during the 3 years. In future evaluations, it would be ideal to include ongoing cost data reporting requirements. Second, data were obtained from a convenience sample subset of PCNASP-funded state health departments and, similarly, state health department–reported costs for a subset of their partners. Because of this small sample, the variability of populations across states engaged in PCNASP and the age of PCNASP programs, the estimates presented are not generalizable to the broader population of stroke care and QI program partners. Lastly, the parameters defining types of resources and efforts, which should be included or excluded when reporting in-kind contributions from partners, were not specified in the instructions for the data collection tool. Furthermore, we were not able to validate what was reported as in-kind and whether it was directly associated with achieving the aims and objectives of PCNASP and not associated with other non-PCNASP stroke initiatives.

This study highlights the costs of implementing components of stroke systems of care at the state level among 6 states. Past implementation literature is limited and reflects only selected interventions, rather than comprehensive QI initiatives (32). Our study is the first to document the costs incurred by state health departments implementing stroke systems of care across multiple programs. Results can guide future program budgets, strategies, and focused interventions; improve planning for sustainability; and increase the potential scale and adoption of programs across the country. On average, 795,000 Americans annually have a stroke, at a cost of nearly $40.1 billion per year (1), which highlights an opportunity to make investments in statewide systems of care through programs like PCNASP. On a small scale, identifying the estimated costs for public health and the health care sector to establish and implement components of statewide systems of care can help policy makers, public health, and medical officials of the potential cost effectiveness to implement and sustain efforts, such as the PCNASP, that aim to reduce the burden of stroke.
